# Surgical management of superior sulcus tumors: A twenty-year experience of an oncological high volume referral centre

**DOI:** 10.3389/fonc.2022.1080765

**Published:** 2023-01-12

**Authors:** Luca Bertolaccini, Monica Casiraghi, Domenico Galetta, Francesco Petrella, Antonio Mazzella, Giorgio Lo Iacono, Lara Girelli, Claudia Bardoni, Shehab Mohamed, Valeria Musso, Giulia Sedda, Lorenzo Spaggiari

**Affiliations:** ^1^ Department of Thoracic Surgery, IEO, European Institute of Oncology IRCCS, Milan, Italy; ^2^ Department of Oncology and Hemato-Oncology, University of Milan, Milan, Italy

**Keywords:** superior sulcus tumour, lung cancer, pancoast, survival, biostatistic analysis

## Abstract

**Objectives:**

Superior sulcus tumour, which affects the lung’s apex, is an uncommon subtype of non-small cell lung cancer (NSCLC). The current study examined the clinical characteristics and management of superior sulcus NSCLC patients in a high-volume referral oncological centre over 22 years.

**Methods:**

Retrospective review of 100 surgeries with curative intent for superior sulcus NSCLC over 22 years (July 1998 – December 2020). The surgical approach was defined according to the lesion site and the anatomy of the thoracic inlet. Survival curves, including non-cancer-related deaths, were drawn using the Kaplan-Meier methods, and the log-rank test was used to evaluate differences in survival across groups of patients. Cox proportional hazards regression was used to assess the association between selected clinical and pathologic characteristics on OS.

**Results:**

54 patients received induction treatments. The surgical approach was anterior thoracotomy in 53 patients, Paulson incision in 30, and a combined in 8. The median postoperative length of stay was 11 days (range: 5 – 27 days). Overall 90-day mortality was 6.93%. The median OS was 24.3 months. After a median follow-up of 3 years, 5-year and 10-year OS rates were 33.9% and 26.4%, respectively. A significantly lower 5-year OS was observed in patients with the nodal disease (46.6% in pN0 vs 13.2% in pN+; p = 0.024), without preoperative treatments (41.0% in patients without preoperative treatments versus 17.4%; p = 0.09) and anteriorly located tumour (anterior vs posterior: 17.4% vs 49.1%; p = 0.032). Cox proportional hazards regression showed better survival in the pT1 stage (HR = 4.6; 95% CI: 1.9 – 11.2; p = 0.00076) and in R0 (HR = 4.2; 95% CI: 1.4 – 12.5; p = 0.010).

**Conclusions:**

Superior sulcus tumours still represent a life-threatening condition that, while curable in a significant proportion of cases, requires complex procedures with high surgical risks and a multimodality treatment setting. An optimal surgical approach should be planned to maximise resection completeness and survival. Other factors affecting survival are related to tumour staging, emphasising the importance of a meticulous preoperative workup and candidate selection to identify those expected to benefit from a survival benefit.

## Introduction

The superior sulcus tumour, which affects the lung’s apex, is an uncommon subtype of non-small cell lung cancer (NSCLC) (1). In 1924 and 1932, the Philadelphia radiologist Pancoast initially characterised this tumour, developing at the chest apex, as carcinomas of uncertain origin (2). Before the 1950s, the superior sulcus tumour was considered incurable. Chardack and Maccallum employed preoperative irradiation followed by surgery for the first time (3). In 1961, it was determined that preoperative radiation followed by surgery increased the 5-year survival rate. In the 1990s, two comparable prospective studies by the Southwest Cancer Research Group/North American study group (SWOG9416/Intergroup 0160) (4) and the Japan Clinical Oncology Group (JCOG 9806) (5) demonstrated that preoperative chemoradiotherapy followed by surgery was associated with a higher rate of survival. The location of the lesion and the architecture of the thoracic inlet determined the surgical strategy. The anterior approach (transmanubrial approach + anterolateral thoracotomy or hemiclamshell incision) for the superior sulcus entering the anterior thoracic inlet (subclavian vessels, first rib, distal part of innominate vein) was described by Grunenwald and Spaggiari in 1997 (6). The posterior technique (traditional Paulson posterior thoracotomy) was used for treating posterior thoracic inlet-invading malignancies (7). The combined approach (anterior and lateral or posterolateral thoracotomy) was based on the severity of middle and posterior inlet involvement (8).

The current study examined the clinical characteristics and management of patients with superior sulcus NSCLC treated in a high-volume comprehensive cancer centre over 22 years.

## Material and methods

The Ethics Committee and the Internal Review Board, informed of the database extraction, did not require approval because of the study’s retrospective nature. This manuscript was written according to the Strengthening the Reporting of Cohort Studies in Surgery (STROCSS) Statement (9). The STROCSS checklist is available as **Supplemental File 1**.

We reviewed the clinical records of 100 patients who received curative surgery for superior sulcus NSCLC at our institution over 22 years (July 1998 – December 2020). Before surgery or medical treatment, patients obtained written authorisation at the time of hospital admission to use their personal information for therapeutic purposes and separately for epidemiologic research investigations. All patients with an apical tumour and Pancoast syndrome or superior sulcus tumours with invasion of the chest wall, vertebral body, or subclavian arteries as detected by computed tomography (CT) or magnetic resonance imaging were included, according to a previous study (10). All patients received a clinical history, physical examination, routine blood tests, electrocardiogram, spirometry, and perfusion lung scan before surgery. Chest radiography, bronchoscopy, whole-body CT scan, and 18-fluoro-deoxyglucose positron emission tomography (PET) whole-body scan were included in the staging strategy for all patients. Standard angiography or magnetic resonance imaging was not employed routinely and was only conducted on a restricted group of patients. Mediastinoscopy or endobronchial ultrasound – transbronchial needle aspiration (EBUS – TBNA) was performed in cases of suspected N2 illness based on CT evidence of enlarged mediastinal nodes (>1.5 cm) or pathologic PET uptake. If pN2 disease was verified, neoadjuvant chemotherapy or chemoradiation was delivered, and surgery was considered following an objective response or absence of disease progression. As a result of the fact that some of the patients got radiotherapy or chemotherapy under the care of the referring physicians, no uniform procedure was implemented. When mediastinoscopy or EBUS – TBNA excluded concomitant N2 disease, patients with pathologic ipsilateral supraclavicular lymph node disease (N3) were evaluated for excision (after induction chemotherapy or chemoradiotherapy). We favoured a surgical approach followed by adjuvant therapies, assuming that the tumours were technically amenable to radical resection and that the tests as mentioned above ruled out N2 or N3 lymph node involvement. In contrast, in the case of a substantial mass of N+ disease, all surgical resection candidates received at least three cycles of induction chemotherapy, alone or in conjunction with radiotherapy (<45 Gy), followed by re-evaluation with a whole-body CT scan and PET scan. The location of the lesion and the architecture of the thoracic inlet determined the surgical strategy. Standard pulmonary resections (lobectomy and/or sublobar resections) were performed only in severe respiratory impairment or considerable comorbidity cases. Routinely, systematic mediastinal lymph node dissection was performed. Chest wall resection was performed concurrently with lung parenchyma.

### Statistical analysis

The mean and standard deviation (SD) of quantitative variables were used, while nominal variables were presented as the presence or absence of the occurrence. The Kruskal–Wallis rank test was employed for continuous variables, and for categorical variables, the Fisher exact test was used. The time gap between operation and death was defined as the OS. The time interval between resection and disease relapse was defined as recurrence-free survival (RFS), and patients without recurrence were censored at the latest time known to be recurrence-free. The reverse Kaplan–Meier approach calculated the median OS and RFS. The median OS, hazard ratio (HR), and 95% confidence intervals (CI) were used to describe differences in survival rates, and the log-rank test was used to compare them. Bonferroni correction was applied for multiple comparisons, and a p-value of less than 0.05 was considered significant. The *standard*, *EZR*, *irr*, and *rcmdr* packages were used in RStudio (R version 4.2.1, Funny-Looking Kid) for statistical analysis (11, 12).

## Results

One hundred patients with potentially resectable superior sulcus NSCLC were operated (**Table 1**). Eighty-five patients were men (85%), and 15 were women. 96% were smokers or former smokers. The median age was 62 years (range: 44 – 80). 9 patients (9%) had significant comorbidities (chronic ischemic heart disease in 6, limited pulmonary function in 3). 54 (54%) patients received induction treatments (43 had chemotherapy, 9 had chemoradiotherapy, and two had radiotherapy).

**Table 1:** Demographics and clinical characteristics of the patients with potentially resectable superior sulcus NSCLC.

Characteristics	No. (%)
Male/Female ratio	5.67
Age, median (range)	62 (44 – 80)
Comorbidities * Chronic ischemic heart disease * Limited pulmonary function	9 (9) 6 (6) 3 (3)
Induction treatments * Chemotherapy * Chemoradiotherapy * Radiotherapy	54 (54) 43 (43) 9 (9) 2 (2)
Surgical approaches * Anterior compratement - Hemiclamshell incision - Transmanubrial approach + anterolateral thoracotomy - Transmanubrial approach + hemiclamshell incision - Transmanubrial approach alone * Posterior compartment (Paulson approach) * Combined mixed transmanubrial approach and Paulson incision	52 (51.5) 21 (21) 18 (18) 7 (7) 7 (7) 38 (38) 10 (10)
Lung resections * Lobectomies or bilobectomies * Lobectomies with bronchoplastic reconstruction * Non-anatomical segmentectomies * Pneumonectomies	84 (84) 7 (7) 6 (6) 3 (3)

Values are expressed as numbers (percentages) as otherwise defined.

In 52 patients (51.5%), the tumour was situated in the anterior compartment and was approached *via* hemiclamshell incision in 21 (21%), *via* transmanubrial approach + anterolateral thoracotomy in 18 (18%), *via* transmanubrial approach + hemiclamshell incision in 7 (7%), and *via* transmanubrial approach alone in 7 (7%) patients. In 38 patients (38%), the tumour was located in the posterior compartment and was operated on *via* the Paulson approach. The remaining ten patients (10%) presented with a tumour occupying the whole apex of the thorax, thus requiring a combined mixed transmanubrial approach and Paulson incision. The types of lung resections included 84 lobectomies or bilobectomies (84%), seven lobectomies with bronchoplastic reconstruction (bronchial sleeve) (7%), 6 non-anatomical segmentectomies (6%), and three pneumonectomies (3%). In 98 (98%) patients, nodal dissection was systematic. Twenty-three patients required an associated vascular resection (23%). The postoperative course was uneventful in 46 patients (46%). In 35 patients (35%), minor complications occurred. Twenty patients (20%) experienced a major complication. In 11 patients, a reoperation was needed (11%). The median postoperative length of stay was 11 days (range: 5 – 27 days). Overall 90-day mortality was 6.93%. All patients were reviewed according to the VIII TNM staging system (13). Sixty-five patients (65%) were staged pT3. Fourteen patients (14%) were T4 stage. Lymph node involvement was present in 32 patients (32%): N1 (ipsilateral hilar lymph node involvement) in 17 (17%) patients, N2 (ipsilateral mediastinal lymph node involvement) in 13 (13%), and N3 (supraclavicular or contralateral hilar/mediastinal lymph node) in 4 (4%); 2 (2%) patients were Nx. A pathologically radical resection (R0) was achieved in 85 patients (85%). 38 (38%) received postoperative treatments: radiotherapy in 33 (33%) patients, chemoradiotherapy in 4 (4%), and chemotherapy in 1 (1%) patient.

**Figure 1:**
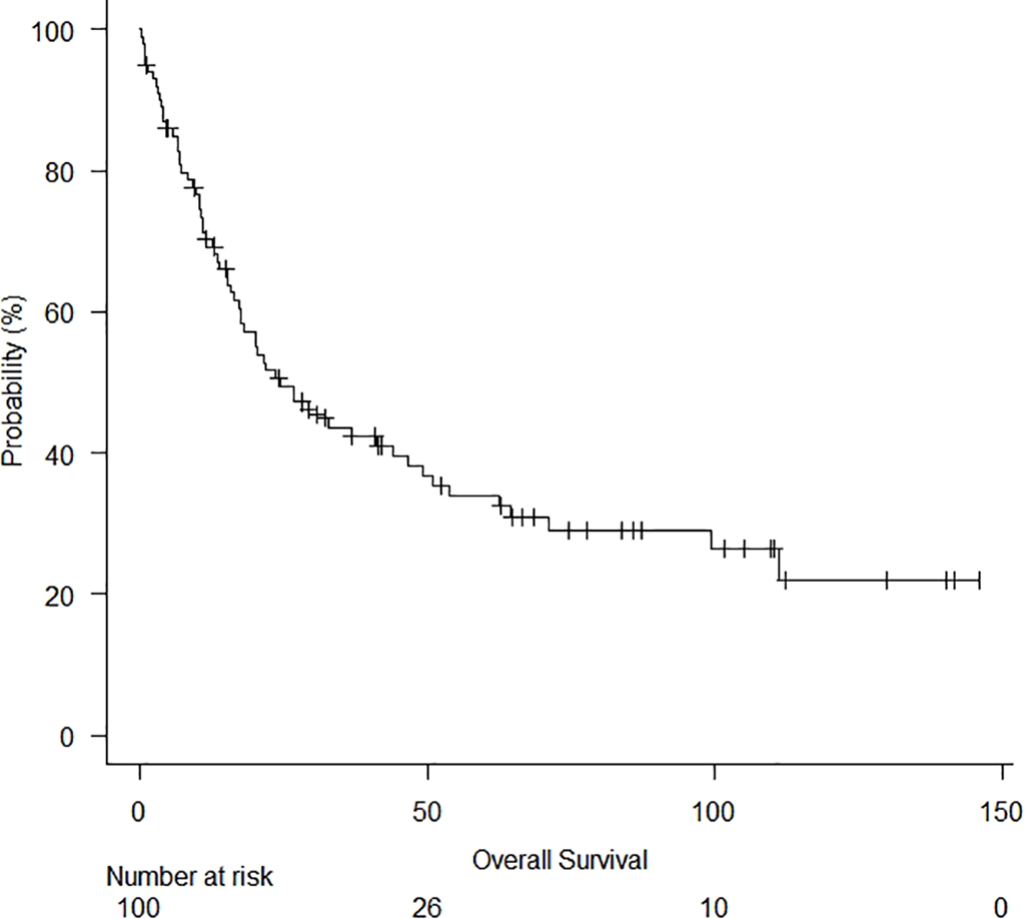
Overall survival (median OS was 24.3 months).

**Figure 2:**
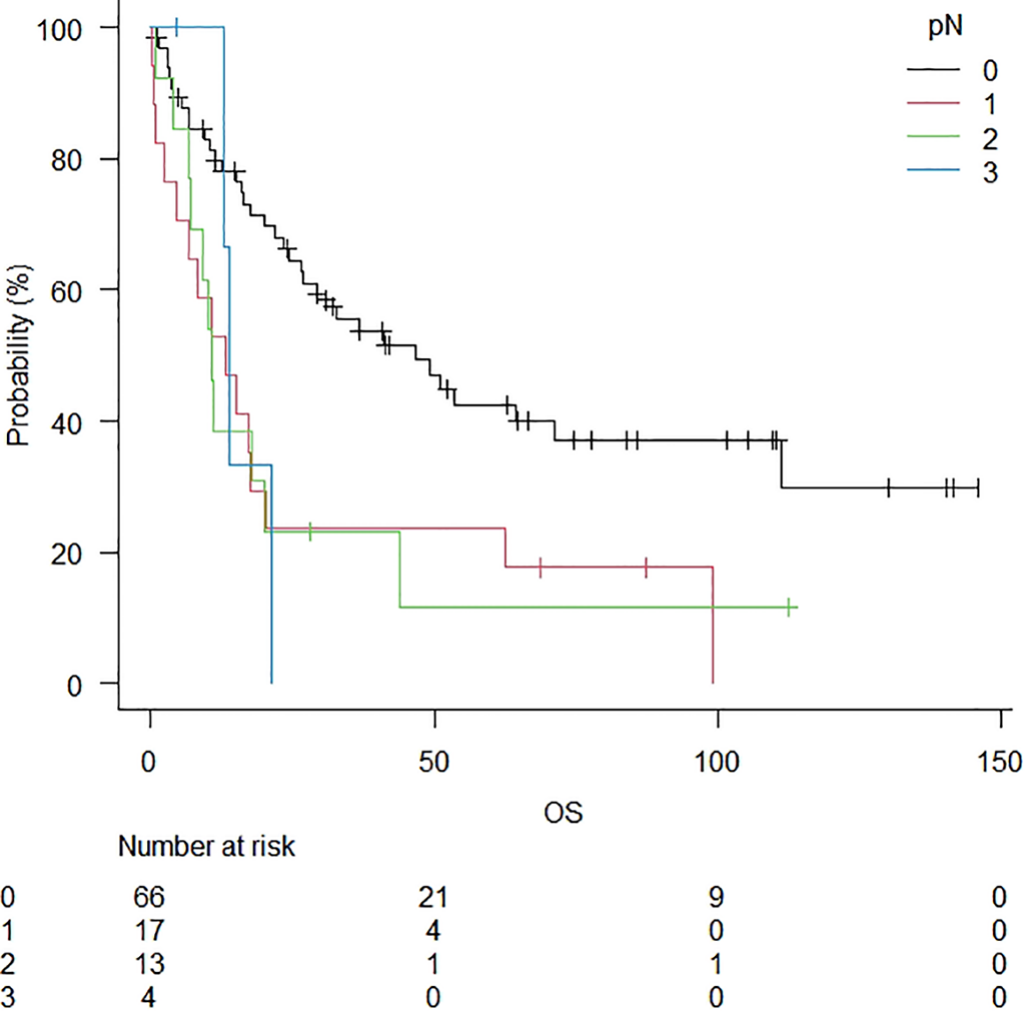
Overall survival in patients stratified for lymph node disease. A significantly lower 5-year OS was observed in patients with the nodal disease (46.6% in pN0 vs 13.2% in pN+; p = 0.024).

After the study, 32 patients were alive (32%), and 4 had tumour relapse. The minimum duration of observation was 6 months (range: 0 – 146 months, median follow-up: 36 months). The median OS was 24.3 months (**Figure 1**). After a median follow-up of 3 years, 5-year and 10-year OS rates were 33.9% and 26.4%, respectively (**Table 2**). A significantly lower 5-year OS was observed in patients with nodal disease (46.6% in pN0 vs 13.2% in pN+; p = 0.024) (**Figure 2**). The Log-rank trend test showed statistically significant (p = 0.00065). A lower 5-year OS was observed in patients without preoperative treatments, even if not statistically significant (41.0% in patients without preoperative treatments versus 17.4%; p = 0.09). A significantly lower 5-year OS was observed in patients with anteriorly located tumours (anterior vs posterior: 17.4% vs 49.1%; p = 0.032). The Log-rank trend test showed statistically significant (p = 0.03). Cox proportional hazards regression showed a better survival in pT1 stage (HR = 4.6; 95%CI: 1.9 – 11.2; p = 0.00076) and in R0 (HR=4.2; 95%CI: 1.4 – 12.5; p = 0.010). The Log-rank trend test showed statistically significant (p = 0.01).

**Table 2:** Survival analysis.

Overall survival (OS)		p-value
**Median OS**	24.3 months	
**• 5-years OS**	33.9%	
**• 10-year OS**	26.4%	
5-year OS in nodal disease		
**• pNO**	46.6%	p=0.024
**• pN+**	13.2%	
5-year OS according to location		
**• Anterior**	17.4%	p=0.032
**• Posterior**	49.1%	

## Discussion

A superior sulcus tumour is positioned at the apex of the lung and has the radiographic invasion of the first rib or apical chest wall components. Based on two unique prospective trials, the usual treatment for superior sulcus tumours is induction chemoradiotherapy followed by surgery. Due to the technical difficulties related to the anatomical features and routes to the thorax, attaining complete excision of the superior sulcus tumour is a significant surgical task. Patients receive *en bloc* excision of the affected lung and chest wall *via* posterolateral thoracotomy and a wide incision. The transmanubrial approach is the most effective method for visualising the apical features of anterior sulcus tumours. Typically, a second thoracotomy is required for lung resection, resulting in pain and subsequent problems. After surgery, the morbidity and death rates for superior sulcus tumour range from 10 to 55% and 0 to 7%, respectively (14).

The 5-year survival rate for superior sulcus tumour patients has grown significantly over the past century due to the evolution of treatment options (4). Notwithstanding, a significant number of patients perished because of metastasis and recurrence. Due to the restricted number of patients, data on the long-term survival of superior sulcus tumours were scarce. Nevertheless, a few papers explored the prognostic characteristics of superior sulcus tumour patients, which might differentiate individuals into a unique category, and proposed a more aggressive treatment. Four prognostic markers were identified: surgical margin status, pathologic response, T stage, and lymph node status. Superior sulcus tumour patients are often diagnosed in the T3 or T4 stages (1, 15).

En bloc resection has been the primary strategy for treating superior sulcus tumours since the turn of the twentieth century. Positive surgical margins become possible sources of local recurrence due to the presence of tumour cells. Pathologic complete response can be achieved in approximately 20% of patients after preoperative chemoradiotherapy, which was previously believed to be a prognostic indicator. The tumours of T4 individuals are more prone to infiltrate surrounding tissue. In addition, a more significant prevalence of positive surgical margins is reported in T4 patients, which may result in worse survival rates for superior sulcus tumour patients. The state of lymph nodes is another crucial prognostic factor. In the positive mediastinal lymph node field, postoperative radiation is advised for stage IIIa N2 NSCLC patients.

Nevertheless, only a handful of the included publications validated postoperative irradiation for superior sulcus tumour patients with a positive surgical margin or N2 metastatic disease. On certain patients, further harsh treatment procedures may be administered. Due to the invasion of neighbouring tissues or organs, total excision of the superior sulcus tumour is challenging, although preoperative chemoradiotherapy has been shown to increase the complete resection rate (1).

Therefore, the T stage and the response to preoperative neoadjuvant therapy are significant determinants of complete resection. After surgery, the Southwest Oncology – lead Intergroup prospective phase 2 trial (SWOG-9416/INT-0160) attempted to add two cycles of consolidation chemotherapy based on etoposide and cisplatin (16). However, patients were unable to complete the adjuvant treatment prescribed to them. The Southwest Oncology Group (SWOG) S0220 trial included two cycles of docetaxel-based consolidation chemotherapy (17). Although R0 and local control rates have improved, distant metastases, particularly brain metastases, pose a grave threat to patient survival.

The JCOG 9806 trial showed a 56% 5-year survival rate, much greater than the average of 30%. Unfortunately, only surgical patients were included in these reported investigations (5).

Additionally, surgical procedures have evolved during the past two decades (15). Various highly effective approaches for anterior Pancoast tumour types with vascular involvement and the management of invasion of the vertebral column have been developed, allowing for the standard *en bloc* resection of tumours along with the involved adjacent structures (18).

### Limitations

This article has several limitations. Due to the small number of patients with superior sulcus tumours, most research has required more time to collect sufficient patient data. Due to the paucity of relevant research published, the duration of the considered studies was also extensive. Therefore, different patients may be diagnosed and treated differently.

Due to the limited number of investigations, most studies had a lengthy period, and the staging version was not clearly stated. Consequently, subgroup analysis was not possible.

## Conclusions

Superior sulcus tumours still represent a life-threatening condition that, while curable in a significant proportion of cases, requires complex procedures with high surgical risks and a multimodality treatment setting. An optimal surgical approach should be planned to maximise resection completeness and survival. Other factors affecting survival are related to tumour staging, emphasising the importance of a meticulous preoperative workup and candidate selection to identify those expected to benefit from a survival benefit.

## Data availability statement

The data analyzed in this study is subject to the following licenses/restrictions: The datasets generated during and/or analysed during the current study are not publicly available but are available from the corresponding author on a reasonable request. Requests to access these datasets should be directed to luca.bertolaccini@gmail.com.

## Ethics statement

Ethical review and approval was not required for the study on human participants in accordance with the local legislation and institutional requirements. The patients/participants provided their written informed consent to participate in this study.

## Author contributions

LB and LS contributed to the conception and design of the study. GS organized the database. LB performed the statistical analysis. LB wrote the first draft of the manuscript. MC, DG, and FP wrote sections of the manuscript. All authors contributed to the manuscript revision, read, and approved the submitted version.
